# Influenza A (H1N1) virus infection complicated by checkpoint inhibitor pneumonitis: A case report

**DOI:** 10.1097/MD.0000000000051109

**Published:** 2026-10-16

**Authors:** Yifan Cheng, Huiying Zhao, Youzhong An

**Affiliations:** a Department of Critical Care Medicine, Beijing Jishuitan Hospital, Capital Medical University, Beijing, People’s Republic of China; b Department of Critical Care Medicine, Peking University People’s Hospital, Beijing, People’s Republic of China.

**Keywords:** checkpoint inhibitor pneumonitis, glucocorticoids, influenza A (H1N1) virus infection

## Abstract

**Rationale:**

Checkpoint inhibitor pneumonitis (CIP) is a common, potentially severe adverse event in cancer patients receiving neoadjuvant immunotherapy, with glucocorticoids as the standard first-line treatment. Traditionally, glucocorticoids are avoided in confirmed viral pneumonia because they may delay viral clearance and increase secondary infection risk. No standardized strategy is available for managing concurrent CIP and viral pneumonia, and inappropriate treatment can lead to irreversible clinical deterioration.

**Patient concerns:**

Following neoadjuvant immunotherapy, the patient developed concurrent influenza A (H1N1) infection and CIP, accompanied by persistent respiratory dysfunction and progressive pulmonary lesions.

**Diagnoses:**

Combined with the patient’s immunotherapy history, respiratory symptoms, imaging changes, and positive influenza A (H1N1) etiological results, a final diagnosis of concurrent CIP and H1N1 viral pneumonia was established.

**Interventions:**

The patient initially received antiviral and empirical antibiotic therapy. Due to ongoing clinical deterioration and poor response to anti-infective treatment, glucocorticoids were reinitiated to target CIP after comprehensive evaluation.

**Outcomes:**

The patient showed early radiographic worsening and intractable respiratory failure with no response to initial anti-infective management. After glucocorticoid administration, clinical symptoms and pulmonary lesions improved significantly, achieving a favorable recovery.

**Lessons:**

Isolated antiviral and antibiotic treatment may fail to control disease progression in patients with concurrent CIP and influenza A pneumonia. Timely and rational glucocorticoid use can reverse clinical deterioration and improve prognosis even in the setting of active viral infection. This case provides practical evidence for the individualized management of overlapping CIP and viral pneumonia.

## 1. Introduction

Identifying the etiology of severe pneumonia is critical for its diagnosis and treatment. In intensive care units, severe pneumonia often involves mixed pathogens, necessitating a diverse array of diagnostic and therapeutic modalities, including empiric use of broad-spectrum antibiotics and antiviral agents. Nevertheless, some cases of pneumonia present with less apparent causes. Checkpoint inhibitor pneumonitis (CIP) is a serious and potentially fatal complication of immune checkpoint inhibitor (ICI) therapy.^[1,2]^ Diagnosis of CIP relies on a comprehensive assessment, including ICI exposure history, new pulmonary infiltrates, changes in respiratory symptoms, exclusion of alternative causes, and the patient’s response to corticosteroid treatment.^[3,4]^ Due to its atypical presentation, CIP can be easily overlooked, particularly in patients with a known infectious pathogen. Furthermore, in some cases where patients are co-infected with other pathogens, even if CIP is diagnosed, its treatment regimen may conflict with the existing treatment plan. Consequently, critical care physicians face numerous hurdles in accurately diagnosing and effectively treating severe pneumonia, which, if overcome, can substantially enhance patient outcomes. Here, we report the case of a patient with non-small cell lung cancer receiving neoadjuvant therapy who developed H1N1 influenza complicated by CIP.

## 2. Patient concerns

A 74-year-old Han Chinese man was admitted to our department with intermittent fever that persisted for several weeks during neoadjuvant therapy. One month earlier, he had been diagnosed with non-small cell lung cancer (cT3N0Mx, stage IIB), with adenocarcinoma strongly suspected as the specific histological subtype. Immunohistochemical analysis of the tumor tissue revealed TTF–1 (+), Napsin A (+), ALK–Ventana (D5F3) (−), CgA (−), Syn (−), CD56 (−), CK5/6 (−), p63 (+), Ki–67 (30%+), and PD–L1 (SP263: TC 0%+). A KRAS exon 2 (G12C) mutation was also identified. The attendig physician initiated neoadjuvant therapy in accordance with prevailing clinical practice guidelines, comprising pemetrexed (800 mg administered on day 1), carboplatin (400 mg on day 1), and pembrolizumab (200 mg on day 1).^[5]^ Nine days after the initial presentation, the patient developed a fever. After several days without improvement, he was admitted to our hospital. Diagnostic tests, using nasopharyngeal swabs for COVID-19 nucleic acid and assays for respiratory viruses, including influenza A and B, respiratory syncytial virus, Mycoplasma pneumoniae (MP), adenovirus and human rhinovirus (HRV), were negative. Cultures of sputum and airway secretions were also negative. The medical team initiated a therapeutic regimen comprising antipyretics, anti-infectives, enoxaparin, and electrolyte and albumin supplementation. Two days later, the patient developed dyspnea and tachypnea after defecation. On evaluation, his vital signs were: peripheral capillary oxygen saturation (SpO_2_) of 78% (without oxygen support); blood pressure of 133/65 mm Hg; respiratory rate of 30 breaths/min; and heart rate (HR) of 125 beats per minute (bpm). Given these clinical manifestations, pulmonary embolism was ruled out based on emergency computed tomography pulmonary angiography (CTPA).

The patient was subsequently transferred to the surgical intensive care unit (SICU) to receive specialized care. He reported no prior severe infections and no history of chronic respiratory or cardiac conditions.

Under high-flow oxygen inhalation support (oxygen flow rate: 60 L/min, FiO_2_: 60%), his SpO_2_ improved to 99%, with a blood pressure of 129/62 mm Hg, Respiratory rate of 24 breaths/min, and HR of 110 bpm. Arterial blood gas analysis revealed a pH of 7.429, partial pressure of oxygen (PO_2_) of 99 mm Hg, partial pressure of carbon dioxide (PCO_2_) of 37 mm Hg, lactate (Lac) level of 1 mmol/L, and an oxygenation index of 165 mm Hg (low), suggesting the potential onset of acute respiratory distress syndrome.

Blood tests revealed several abnormalities, including pancytopenia, likely attributable to prior chemotherapy-induced myelosuppression (CIM), followed by a rapid increase in white blood cell (WBC) count, suggesting a new infection, further supported by elevated C-reactive protein levels. Serum chemistry analysis revealed slightly elevated aspartate aminotransferase levels and decreased albumin levels, indicating a consumptive physiological state. Abnormal coagulation parameters were observed in disseminated intravascular coagulation (DIC) tests, with a prothrombin time of 13.3 second, fibrinogen level of 356 mg/dL, activated partial thromboplastin time of 35.1 second, fibrin degradation product (FDP) level of 110.2 μg/mL, and D-dimer level of 15,490 ng/mL (Table 1). These results indicated that the patient was in a hypercoagulable state and suggested an inflammatory condition.

**Table 1:** Blood tests when the patient was transferred to the SICU.

Complete blood count		Blood biochemistry		Blood coagulation function	
**WBC (10**^**9**^**/L**)	**19.2**	**ALT (U/L**)	**18**	**PT (s**)	**13.3**
NEUT% (%)	91.5	AST (U/L)	61	APTT (s)	35.1
LYMPH% (%)	4.5	TP (g/L)	49.1	FIB (mg/dL)	356
MONO% (%)	3.6	ALB (g/L)	29	FDP (µg/mL)	110.2
RBC (10^12^/L)	3.74	TBIL (µmol/L)	13.9	D-dimer (ng/mL)	15,490
Hb (g/L)	118	DBIL (µmol/L)	3.9		
HCT (%)	33.9	SCr (µmol/L)	71		
PLT (10^9^/L)	103	BUN (mmol/L)	5.7		
CRP (mg/L)	234.7	UA (µmol/L)	170		
		GLU (mmol/L)	5.71		

Physical examination revealed moist rales in both lungs, more pronounced in the lower lung fields bilaterally.

Serial chest X-rays conducted after the patient was transferred to the SICU revealed progression in the severity of the bronchovascular and patchy opacities compared to the previous imaging findings obtained prior to the patient’s admission (Fig. 1). Furthermore, a chest computed tomography (CT) (Fig. 2) showed posttreatment changes in the right upper lobe, consistent with lung cancer, along with interstitial abnormalities in both lungs and the presence of pleural effusion.

**Figure 1.**
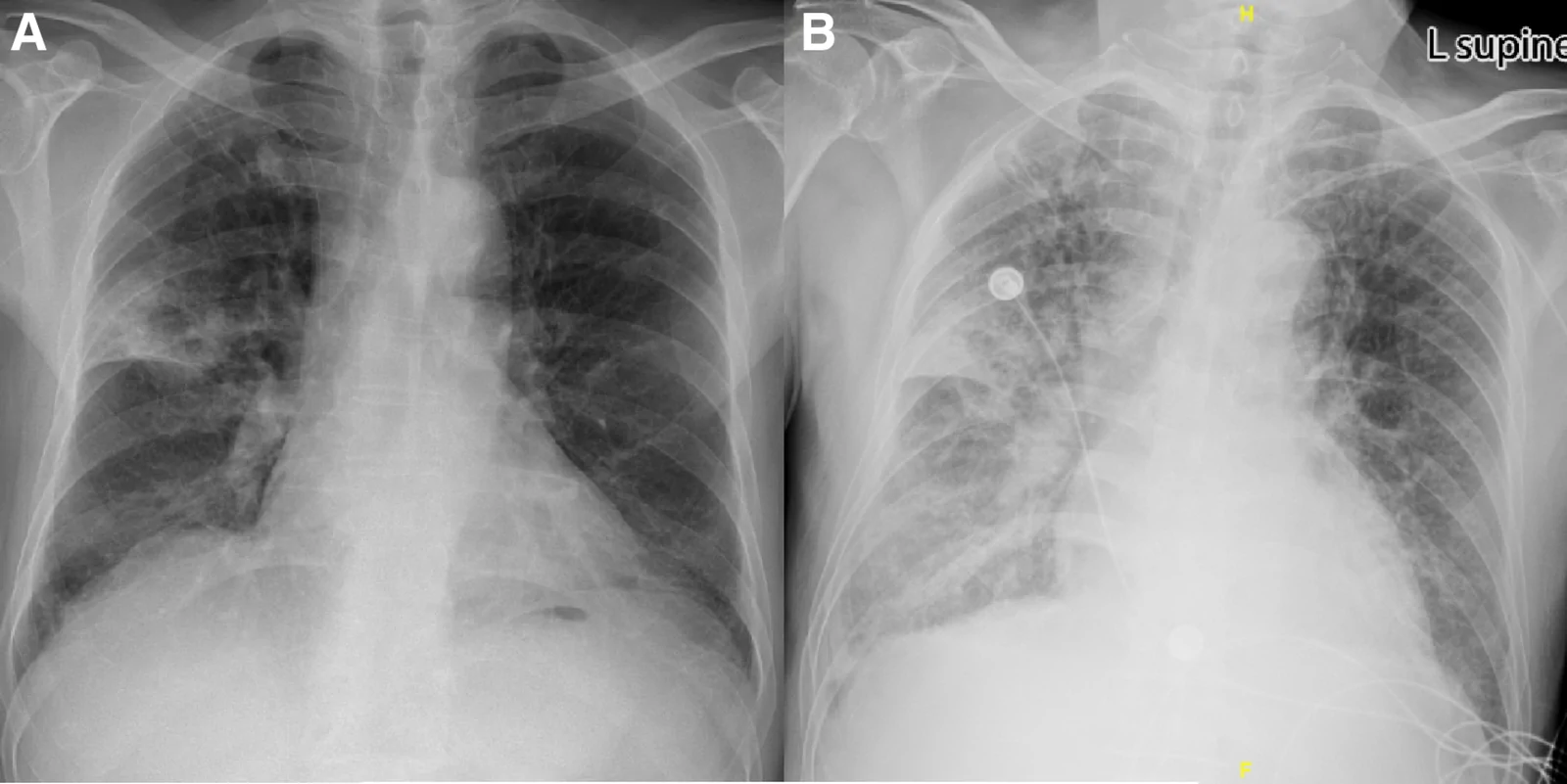
Chest X-ray showing increased pulmonary exudates and right pleural effusion (B) compared to previous findings (A).

**Figure 2.**
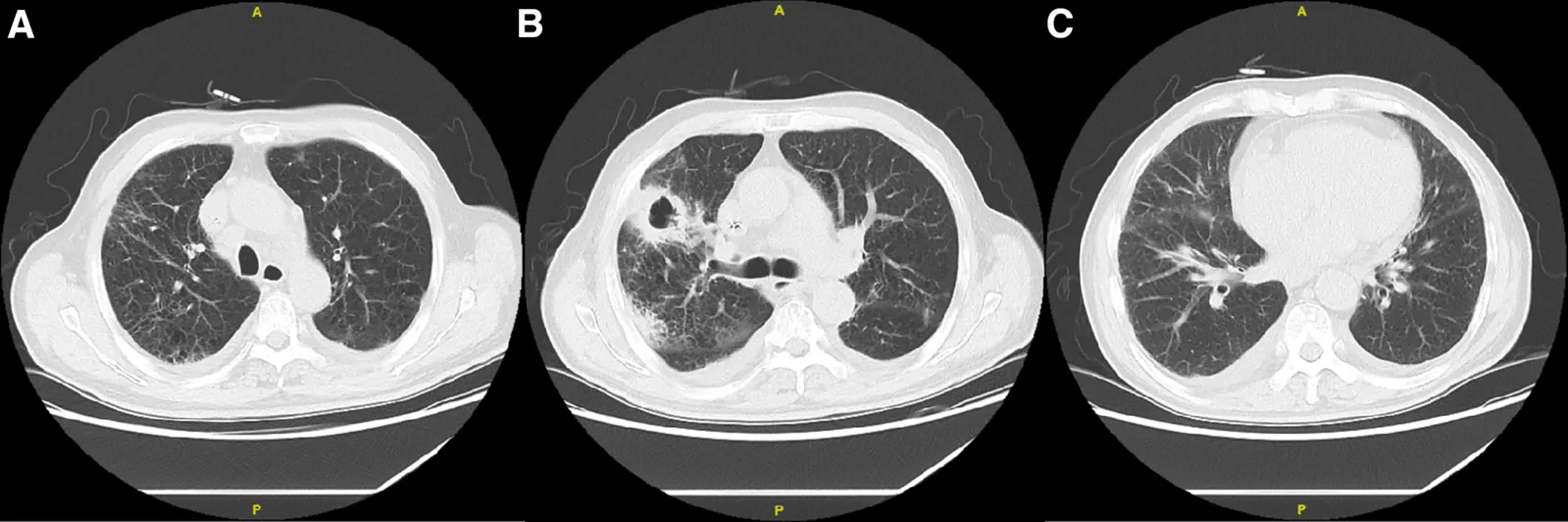
Chest computed tomography (CT) scan (A–C) showing the presence of lung cancer (posttreatment) in the anterior segment of the right upper lobe. The lesion remained predominantly stable; however, the cavity enlarged compared with previous imaging studies. This was accompanied by localized inflammatory changes around the lesion and interstitial lung disease affecting both lungs. Furthermore, bilateral pleural effusions were noted to be increased compared with previous radiological assessments.

At this point, the negative pathogen test results, combined with the radiological changes, ineffective antiviral treatment, and history of neoadjuvant therapy, led to the diagnosis of CIP. Subsequently, glucocorticoid therapy was initiated with methylprednisolone 40 mg every 12 hours to enhance therapeutic efficacy.

Two days after the patient’s transfer, repeat blood testing was positive for influenza A (H1N1), which accounted for most of the symptoms and laboratory abnormalities. Consequently, we concluded that the earlier symptoms were caused by an undetected influenza A (H1N1) virus infection. Therefore, we discontinued glucocorticoid therapy and started oseltamivir phosphate according to recommended guidelines.^[6,7]^

Subsequently, the patient received systematic supportive care, including high-flow oxygen inhalation, breathing exercises, atomization, and expectorants for respiratory support; meropenem combined with vancomycin for bacterial infection; oseltamivir phosphate for influenza A (H1N1) infection; anticoagulant therapy for coagulopathy; and nutritional and water-electrolyte support. This comprehensive approach was designed to address the multifaceted aspects of the patient’s condition.

As treatment progressed, the patient’s H1N1 virus remained persistently positive. This was attributed to 2 factors: the compromised immune system and delayed administration of oseltamivir phosphate, which exceeded the recommended 48-hour treatment window. After completing a 23-day oseltamivir phosphate regimen, the viral load became undetectable, and follow-up tests confirmed this negative result 1 day later.

However, despite testing negative for the flu virus, the patient’s clinical improvement was minimal. Persistent respiratory distress was observed, accompanied by radiographic abnormalities (Fig. 3). The patient continued to experience symptoms, including respiratory distress and fever, while inflammatory markers remained abnormal. Chest radiography persistently revealed extensive exudative lesions, and oxygen therapy support could not be reduced; antibiotics and oseltamivir phosphate appeared ineffective.

**Figure 3.**
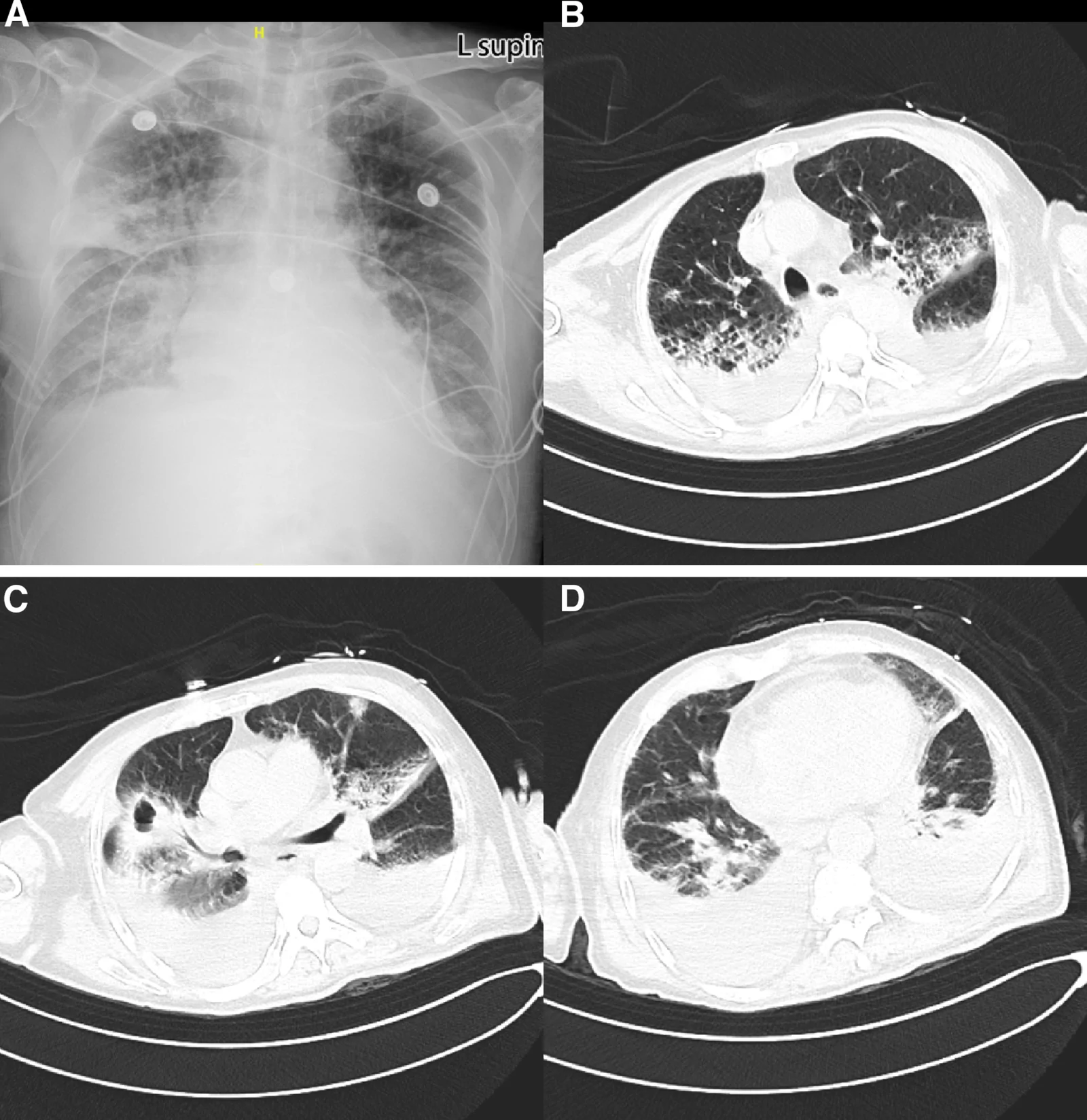
Chest X-ray showing unimproved condition on day 25, despite a negative test for influenza A (H1N1) virus (A). Chest CT scans showing pronounced inflammatory changes in the perilungular regions and pulmonary exudates in both lungs, along with substantial pleural effusion on day 25 when the test for influenza A (H1N1) virus was negative (B–D).

At this stage, the possibility of CIP was reconsidered, and we concluded that the patient had concurrent influenza A (H1N1) infection and CIP. In line with established clinical guidelines, we readministered glucocorticoid therapy, which resulted in significant clinical improvement within a few days. Arterial blood gas analysis showed continuous improvement in the patient’s oxygenation, and laboratory parameters showed positive trends. The abnormal blood test results also reverted to normal levels (Table 2). The patient was afebrile, maintaining an SpO_2_ of 99% without supplemental oxygen. Chest X-ray and CT revealed improvements compared with previous findings, with a reduction in exudative lesions and pleural effusion (Fig. 4). The patient’s general condition was deemed satisfactory, and he was subsequently transferred out of the SICU after 1 week. The patient’s overall treatment timeline is presented in Figure 5.

**Table 2:** Blood tests before being transferred out of the SICU.

Complete blood count		Blood biochemistry		Blood coagulation function	
WBC (10^9^/L)	5.7	ALT (U/L)	9	PT (s)	12.3
NEUT% (%)	76.5	AST (U/L)	16	APTT (s)	29.1
LYMPH% (%)	12.9	TP (g/L)	56	FIB (mg/dL)	207
MONO% (%)	10	ALB (g/L)	34.3	FDP (µg/mL)	2.1
RBC (10^12^/L)	2.53	TBIL (µmol/L)	7.2	D-dimer (ng/mL)	238
Hb (g/L)	87	DBIL (µmol/L)	2.6		
HCT (%)	25.5	SCr (µmol/L)	39		
PLT (10^9^/L)	123	BUN (mmol/L)	4.4		
CRP (mg/L)	1.3	UA (µmol/L)	130		
		GLU (mmol/L)	4.41		

**Figure 4.**
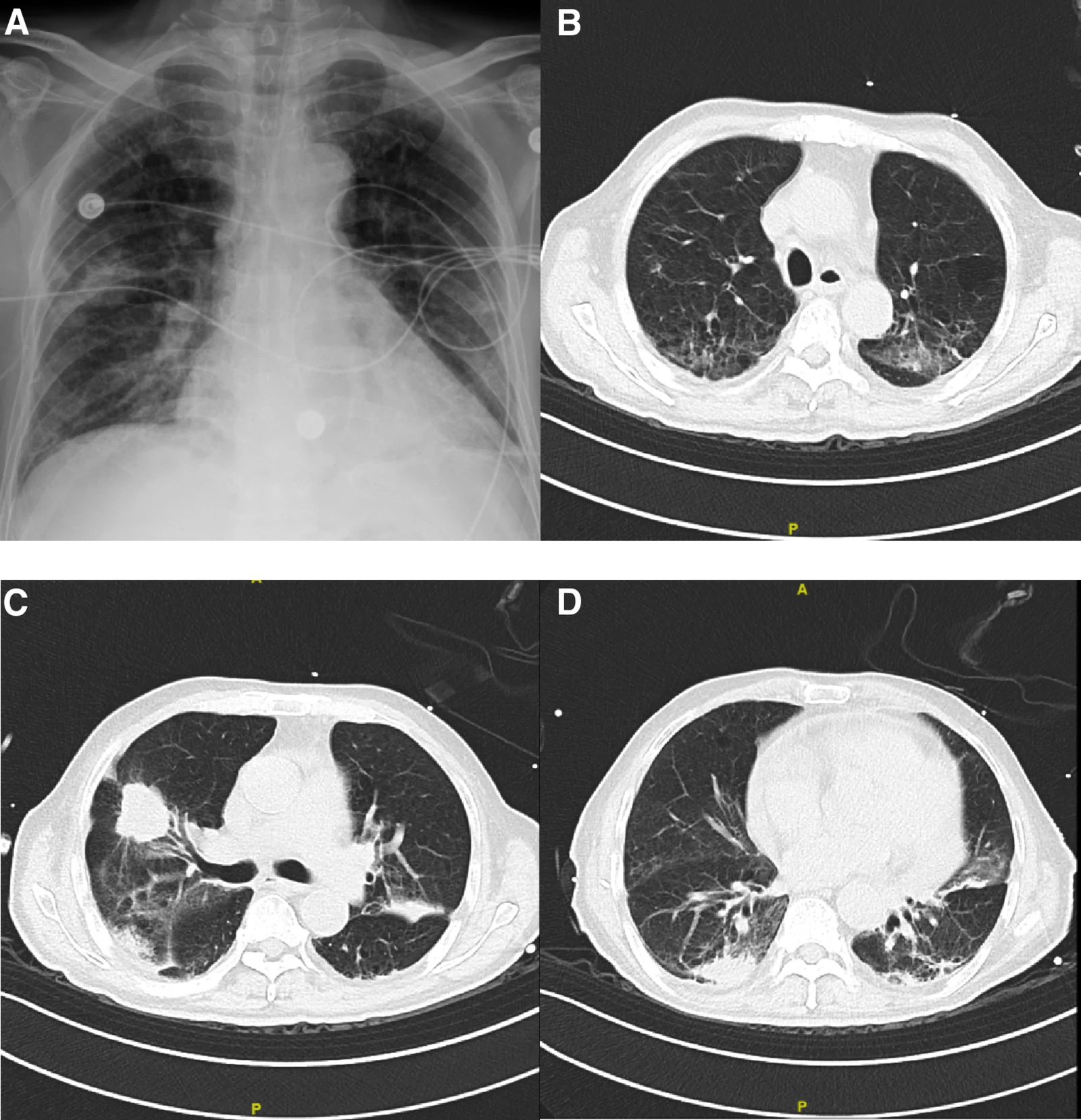
Chest X-ray showing a marked reduction in both pulmonary exudates and pleural effusion (A) was observed following glucocorticoid therapy on day 38. Chest CT scans showed a notable improvement in inflammatory changes, pulmonary exudates, and pleural effusion by day 38 (B–D).

**Figure 5.**
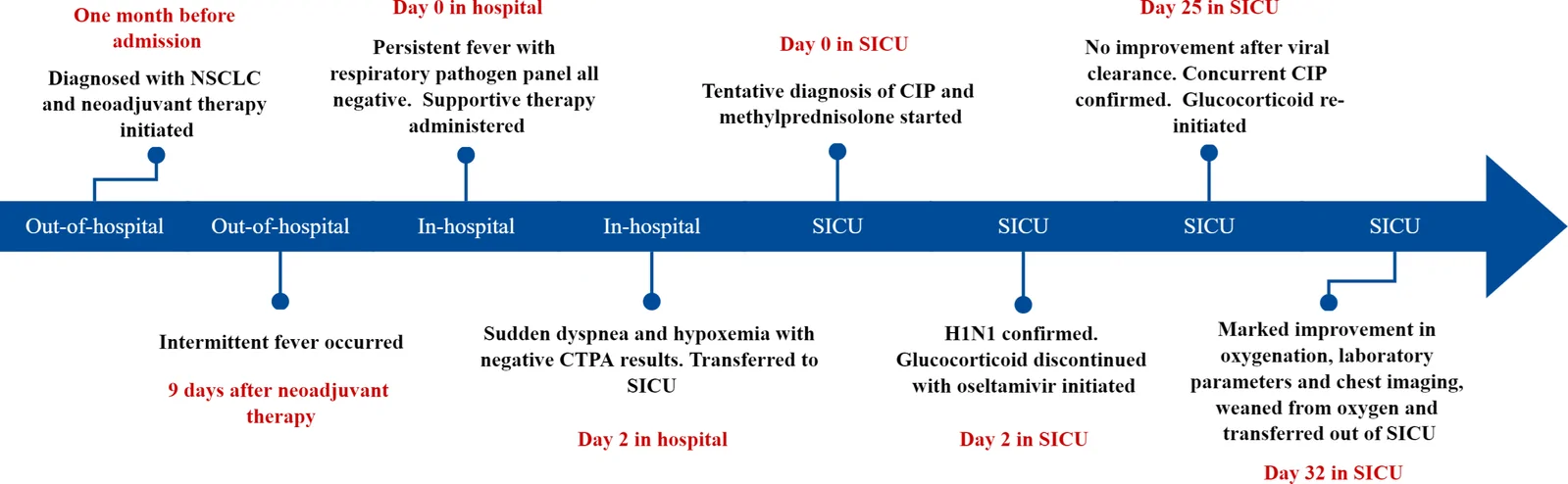
Patient treatment timeline showing the sequence of therapeutic interventions.

## 3. Discussion

Patients in the SICU typically present with more severe disease and dysfunction across multiple organ systems, complicating diagnostic and therapeutic direction. Interventions may have conflicting effects; for instance, the need for airway protection in profound hypoxemia or the use of glucocorticoids to manage infection and fever may inadvertently compromise the immune defenses of critically ill patients and substantially increase the risk of opportunistic infections. In such scenarios, establishing therapeutic priorities, identifying the most critical problems among the myriad of conditions, and mitigating potential complications during systemic therapy are key considerations for intensive care practitioners.

In this present case, while managing the patient’s critical condition, simultaneously addressing other comorbidities, such as coagulopathy and fluid-electrolyte imbalances, was imperative. Furthermore, a timely and comprehensive etiological investigation of severe pneumonia and respiratory distress was essential, necessitating broad screening for various pathogens. Although initial respiratory pathogen tests were negative prior to admission to the SICU, the presence of viral latency, concurrent peak influenza season, and clinical presentation suggested that influenza A (H1N1) infection could not be definitively excluded. Consequently, serial repeat pathogen testing was imperative to ascertain the actual etiology.

After identifying the pathogen and initiating targeted therapy until the patient tested negative for the virus, the patient’s pulmonary symptoms showed no significant improvement. At this stage, the differential diagnosis needed to be expanded to include possible coinfection with other pathogens. Additionally, given the patient’s recent history of neoadjuvant therapy, a differential diagnosis was also needed to account for the potential of CIP.

The diagnostic criteria for CIP include documented exposure to an ICI, development of new radiographic pulmonary infiltrates, exacerbation of preexisting respiratory symptoms or onset of new respiratory symptoms, exclusion of alternative etiologies (such as tumor progression or pulmonary infection), and a demonstrable positive response to corticosteroid therapy in the absence of improvement with antibacterial agents.^[3,4]^

Management of CIP requires a tailored approach based on disease severity. Mild disease (grade 1) may warrant close monitoring with serial follow-up chest CT scans. Moderate CIP (grade 2) typically requires intravenous corticosteroids, such as methylprednisolone, followed by a gradual tapering regimen after symptomatic resolution. For severe CIP (grade ≥3), permanent discontinuation of ICIs should be strongly considered, combined with the intravenous administration of medium-to-high doses of corticosteroids (e.g., methylprednisolone), followed by a prolonged tapering schedule extending beyond 8 weeks. If corticosteroids are ineffective, adjunctive immunoglobulins and/or immunosuppressive agents may be warranted.^[4,8]^

Upon the patient’s admission to the SICU, the negative pathogen tests, documented history of neoadjuvant therapy, and clinical manifestations suggestive of pneumonia raised concern for CIP, and corticosteroid treatment was started. However, after influenza A was detected, corticosteroids were discontinued due to the patient’s chemotherapy-related immunosuppression and elevated infection risk. Continued glucocorticoid use could have increased the likelihood of developing new infections caused by other pathogens. Furthermore, substantial research indicates that glucocorticoids may prolong viral replication time and induce adverse effects.^[9,10]^

A retrospective analysis indicates that the patient was predominantly co-infected with H1N1 and CIP throughout the treatment period. Although glucocorticoids are the first-line treatment for CIP,^[4,8]^ increasing evidence suggests that early use of high-dose corticosteroids is associated with increased mortality rates,^[9,10]^ elevated rates of secondary bacterial infections,^[10]^ and prolonged viral shedding.^[11]^ Notably, research on the combination of glucocorticoids and H1N1 vaccines suggests that these agents may suppress immune responses.^[12]^

Glucocorticoids offer potential therapeutic advantages in the management of influenza A infection, particularly because of their well-documented anti-inflammatory properties. Multiple mechanisms collectively contribute to decreased production of cytotoxic mediators (including IL–6, IL–8, and TNF–α) by pro-inflammatory cytokines during the early phase of the immune response to H1N1 pneumonia.^[13–15]^

Furthermore, a prospective study indicated that adjunctive long-term, low-to-moderate-dose corticosteroid therapy alongside antiviral treatment can mitigate pulmonary damage, lower MOD scores, and decrease hospitalization mortality rates in patients with H1N1 or H5N1 influenza complicated by acute respiratory distress syndrome.^[16]^ Moreover, hematopoietic cell transplant recipients who received high-dose glucocorticoid therapy upon influenza diagnosis had a lower likelihood of requiring mechanical ventilation during H1N1 infection.^[17]^

Recent investigations have also shown that combining systemic corticosteroids with early antiviral medications can prevent progression to severe pneumonia in patients with asthma infected with H1N1.^[18]^ Additionally, some evidence suggests that early glucocorticoid therapy combined with mTOR inhibitors in severe influenza A (H1N1) may improve patient prognosis.^[19]^

Therefore, the role of glucocorticoids in H1N1 influenza infections, alone or in combination with other diseases, remains controversial. Glucocorticoids play an important role in anti-inflammatory therapy, treatment of severe pneumonia, reduction of systemic response, and improvement of outcomes. However, in immunosuppressed patients with confirmed influenza A (H1N1) infection, glucocorticoids can prolong viral shedding and increase the risk of secondary infection. Therefore, in this patient with H1N1 infection complicated by CIP, an important question arises: should glucocorticoids have been administered from the outset? This remains an intriguing and controversial issue.

This case report has some limitations. First, as a single-case study, the limited sample size makes it difficult to generalize the findings and treatment responses to a broader patient population. Second, retrospective data collection may yield incomplete or biased information, impacting the accuracy of the conclusions. Finally, due to resource constraints, some critical laboratory parameters were not longitudinally monitored. Future large-scale, prospective, controlled studies are needed to validate these findings and further elucidate the underlying mechanisms.

## 4. Conclusion

This case report describes the diagnostic and therapeutic process of influenza A (H1N1) virus infection complicated by CIP. Severe pneumonia in the ICU typically has multifactorial etiologies. When the causative pathogen is identified, treatment plans should be developed in accordance with existing guidelines while also accounting for the possibility of coexisting conditions. For instance, in patients undergoing neoadjuvant therapy, the risk of CIP should not be overlooked. Based on this patient’s clinical course and treatment response, the timely initiation of appropriate interventions should be considered to optimize therapeutic benefits. In the present case, glucocorticoid therapy appeared to be a key factor contributing to clinical improvement. This case report serves as an outstanding example, providing valuable references for clinical practice and for establishing relevant treatment regimens. As with several other complex ICU cases, this underscores the need for greater professional proficiency, diagnostic precision, and clinical acumen for ICU physicians.

## Acknowledgments

We thank Dr Chenxiao Hao for her significant assistance in completing and submitting this report.

## Author contributions

**Data curation:** Yifan Cheng.

**Formal analysis:** Yifan Cheng.

**Investigation:** Yifan Cheng.

**Methodology:** Yifan Cheng.

**Project administration:** Yifan Cheng.

**Software:** Yifan Cheng.

**Validation:** Yifan Cheng.

**Visualization:** Yifan Cheng.

**Conceptualization:** Huiying Zhao, Youzhong An.

**Funding acquisition:** Huiying Zhao, Youzhong An.

**Resources:** Huiying Zhao, Youzhong An.

**Supervision:** Huiying Zhao, Youzhong An.

**Writing – original draft:** Yifan Cheng.

**Writing – review & editing:** Huiying Zhao, Youzhong An.
